# Identification of Novel Genetic Variants and Comorbidities Associated With ICD-10-Based Diagnosis of Hypertrophic Cardiomyopathy Using the UK Biobank Cohort

**DOI:** 10.3389/fgene.2022.866042

**Published:** 2022-05-24

**Authors:** Alex Gyftopoulos, Yi-Ju Chen, Libin Wang, Charles H. Williams, Young Wook Chun, Jeffery R. O’Connell, James A. Perry, Charles C. Hong

**Affiliations:** Department of Medicine, University of Maryland School of Medicine, Baltimore, MD, United States

**Keywords:** hypertrophic cardiomyopathy, genome-wide association study, genetic susceptibility loci, UK biobank, ICD-10

## Abstract

**Objectives:** To identify previously unrecognized genetic variants and clinical variables associated with the ICD-10 (International Classification of Diseases 10)-based diagnosis of hypertrophic cardiomyopathy in the UK Biobank cohort.

**Background**: Hypertrophic cardiomyopathy (HCM) is the most common genetic cardiovascular disorder with more than 2000 known mutations in one of eight genes encoding sarcomeric proteins. However, there is considerable variation in disease manifestation, suggesting the role of additional unrecognized contributors, genetic and otherwise. There is substantial interest in the use of real-world data, such as electronic health records to better understand disease mechanisms and discover new treatment strategies, but whether ICD-10-based diagnosis can be used to study HCM genetics is unknown.

**Methods:** In a genome-wide association study (GWAS) using the UK Biobank, we analyzed the genomes of 363 individuals diagnosed with HCM based on ICD-10 coding compared to 7,260 age, ancestry, and sex-matched controls in a 1:20 case:control design. Genetic variants were analyzed by Plink’s firth logistic regression and assessed for association with HCM. We also examined 61 biomarkers and other diagnoses in the 363 HCM cases and matched controls.

**Results:** The prevalence of ICD-10-based diagnosis of HCM in the UK Biobank cohort was 1 in 1,342, suggesting disease assignment based on the two ICD-10 codes underestimates HCM prevalence. In addition, common cardiovascular comorbidities were more prevalent in ICD-10-based HCM cases in comparison to controls. We identified two novel, non-sarcomeric genetic variants in *KMT2C* rs78630626*,* and *PARD3B* rs188937806 that were associated with ICD-10 codes for HCM with genome-wide significance (*p* < 5 x 10^−8^). These are associated with an increased odds ratio (OR) of ∼3.8 for being diagnosed with HCM. Minor allele frequency (MAF) of each variant was >1%.

**Discussion:** Disease assignment based strictly on ICD-10 codes may underestimate HCM prevalence. Individuals with HCM were more frequently diagnosed with several comorbid conditions, such as hypertension, atherosclerotic heart disease, diabetes, and kidney failure, suggesting they may contribute to disease manifestation. This UK Biobank database-based GWAS identified common variants in *KMT2C* and *PARD3B* that are associated with HCM diagnosis, which may represent novel modifier genes. Our study demonstrates the feasibility and limitations of conducting phenotypic and genotypic characterization of HCM based on ICD-10 diagnosis in a large population-based cohort.

## Introduction

Hypertrophic cardiomyopathy (HCM) is a genetic disorder of heart muscle characterized by thickened left ventricular wall and intrinsic cardiac hypertrophy and sarcomere disarray. HCM prevalence is estimated at approximately 1:625-1:344 individuals in the general adult population (Hada et al., 1987; Maron et al., 1995; Maron et al., 1999; Zou et al., 2004). HCM is known to be caused most often by rare pathogenic variants in one of eight genes for sarcomere proteins. However, clinical disease expression varies considerably, even among individuals with identical pathogenic variants. Factors that affect phenotypic expression are largely unknown, although it has been hypothesized that disease modifier genes may play a role in the development of HCM.

There is considerable interest in the use of real-world data, such as electronic health records to better understand disease mechanisms and to discover new treatment strategies (Hemingway et al., 2018). The UK Biobank is a large, ongoing prospective cohort study that recruited 502,682 UK participants between 2006-2010. UK Biobank has compiled extensive health-related records and genetic data from the participants (Bycroft et al., 2018; Ashvetiya et al., 2021; Scalsky et al., 2021). Here, using ICD-10 diagnostic codes as a “real world” surrogate for the definitive diagnosis for HCM, we carried out a GWAS using this database.

Our study identified common variants in *KMT2C* and *PARD3B* that are associated with HCM. These may represent novel modifier genes for the development of HCM. Compared to the control population, individuals with HCM were more frequently diagnosed with several comorbid conditions, such as hypertension, atherosclerotic heart disease, diabetes, and kidney failure, suggesting they may contribute to hypertrophic disease manifestation in genetically susceptible individuals. Our study demonstrates the feasibility and limitations of conducting phenotypic and genotypic characterization of hypertrophic cardiomyopathy based on ICD-10 diagnosis in a large population-based cohort.

## Methods

### Ethical Approval

The present study, which involved de-identified data obtained from the UK Biobank Resource under Application Number 49852, received the proper ethical oversight, including the determination by the University of Maryland, Baltimore Institutional Review Board that the study is not human research (IRB #: HF-00088022).

### Study Population

We carried out a GWAS using the UK Biobank to interrogate the genome for statistically significant associations between single nucleotide polymorphisms (SNPs) and clinical manifestations of HCM. The UK Biobank is an ongoing prospective cohort that recruited over 502,682 UK participants between 2006 and 2010. Participants ranged in age from 37–73 years at the time of recruitment. Health-related records were collected from these participants, including clinical data such as biometrics, biomarkers, and diagnostic codes. The Biobank also contains genetic data, with over 820,000 genotyped SNPs and up to 90 million imputed variants available for most individuals.

### Genome-Wide Association Study

Using data from the UK Biobank Resource on 487,310 subjects with imputed genotypes, we performed quality control by removing those with genetic relatedness exclusions (Data-Field 22018–UKB, https://biobank.ctsu.ox.ac.uk/crystal/field.cgi?id=22018; 1532 subjects), sex chromosome aneuploidy (Data-Field 22019—UKB, https://biobank.ctsu.ox.ac.uk/crystal/field.cgi?id=22019; 651 subjects), mismatch between self-reported sex and genetically determined sex (Data-Field 31—UKB, https://biobank.ctsu.ox.ac.uk/crystal/field.cgi?id=31; Data-Field 22001—UKB, https://biobank.ctsu.ox.ac.uk/crystal/field.cgi?id=22001; 372 subjects), recommended genomic analysis exclusions (Data-Field 22010—UKB, https://biobank.ctsu.ox.ac.uk/crystal/field.cgi?id=22010; 480 subjects), and outliers for heterozygosity or missing rate (Data-Field 22017—UKB, https://biobank.ctsu.ox.ac.uk/crystal/field.cgi?id=22077; 968 subjects).

Cases were defined as having using International Statistical Classification of Diseases and Related Health Problems, 10th edition (ICD-10) diagnostic codes for obstructive hypertrophic cardiomyopathy (I42.1) or other hypertrophic cardiomyopathy (I42.2) as their primary or secondary diagnosis at the time of this analysis (July 2020). The selected set of cases was purged of relatedness by removing one from each related pair in an iterative fashion until no related subjects remained. Relatedness was defined as a kinship coefficient greater than 0.044, which treats third-degree relationships (with a kinship coefficient of 0.0625) as related. Kinship coefficients for all subject pairs greater than 0.044 were provided by the UK Biobank as part of the standard data set. A pool of subjects for the control population was initially generated by removing the cases from the full set. Related subjects in the control population were then removed. For each case, twenty individuals from the control population were selected for comparison matching for age, sex, and ancestry. A total of 7,260 individuals were identified for inclusion as controls in the analysis. The twenty matching control subjects were selected for each case subject using incremental tolerance expansion for age and ancestry. The tolerance for age ranged from 0 (exact match) up to 7 years. Ancestry matching was performed with principal components (PCs) supplied by the UK Biobank. The mathematical distance in a graph plotting the PC1 x PC2 was used to test similarity in ancestry. The “distance” in ancestry tolerance ranged from 2 PC units to a maximum of 80 PC units with PC1 (Supplementary Figure S1), ranging from 0 to +400 and PC2 ranging from −300 to +100 units. Using these tolerances, 20 matching controls were found for every case.

The GWAS was performed with Plink’s firth logistic regression model adjusting for age, sex, and 5 PCs using data supplied by the UK Biobank Resource (Bycroft et al., 2018). We chose firth regression because it has been shown to provide the best combination of control for type I error and power for detection of low-frequency variants (Ma et al., 2013; Wang, 2014; Chang et al., 2015). The HCM cases were analyzed with the 40 million imputed genetic variants provided by the UK Biobank with imputation quality scores greater than 0.70. The analysis included covariates of sex, age, and principal components 1 through 5 to adjust for ancestry. Pre-calculated PC data for the first 40 principal components were supplied by the UK Biobank. Our preliminary analysis showed that only the first 5 PCs had significance with *p*-values less than 0.05. Thus, we used only the first 5 PCs in our GWAS.

UK Biobank contains a wealth of baseline clinical information of participants, including comorbidities, height, weight, body mass index, and basic laboratory values (Bycroft et al., 2018). We examined ICD-10 codes (Table 1), ABO blood type, 5 biometric markers, and 61 biomarkers in 363 HCM cases and 7260 controls (Table 2; Supplementary Table S1). The significance of the association of these conditions and variables with HCM patients in comparison to age, sex, ancestry-matched controls were determined by two-tailed Fisher’s exact test.

**TABLE 1:** ICD-10 diagnoses associated with HCM in UKB.

Diagnosis (ICD-10 code)	Cases (*n* = 363)(%)	Controls (*n* = 7260)(%)	P-value
Ventricular tachycardia (I47.2)	12	0	—
Adjustment and management of cardiac pacemaker (Z45.0)	10	0	—
Mitral valve insufficiency (I34.0)	12	1	—
Atrioventricular block, complete (I44.2)	12	5	—
Supraventricular tachycardia (I47.1)	9	1	—
Chronic renal failure; unspecified (I50.9)	8	1	—
Hypotension; unspecified (I95.9)	5	1	—
Presence of cardiac pacemaker (Z95.0)	23	1	2 x 10^−199^
Congestive heart failure (I50.0)	12	1	2 x 10^−61^
Left ventricular failure (I50.1)	10	1	7 x 10–^44^
Cardiomegaly (I51.7)	25	1	3 x 10^−176^
Palpitations (R00.2)	10	1	7 x 10^−38^
Dizziness and giddiness (R42)	7	1	5 x 10^−18^
Pleural effusion; not elsewhere classified (J90)	8	1	1 x 10^−20^
Dyspnea (R06.0)	13	2	7 x 10^−46^
Sleep apnea (G47.3)	7	2	2 x 10^−14^
Cellulitis of other parts of limb (L03.1)	6	2	3 x 10^−8^
Gastroenteritis and colitis of unspecified origin (A09.9)	6	2	8 x 10–^7^
Pure hypercholesterolemia (E78.0)	34	12	1 x 10^−34^
Lobar pneumonia; unspecified (J18.1)	7	2	1 x 10^−12^
Acute renal failure; unspecified (N17.9)	11	2	4 x 10^−29^
Unstable angina (I20.0)	8	2	8 x 10^−14^
Chronic obstructive pulmonary disease; unspecified (J44.9)	7	2	2 x 10^−9^
Procedure not carried out because of contraindication (Z53.0)	11	3	2 x 10^−20^
Syncope and collapse (R55)	16	3	2 x 10^−39^
Old myocardial infarction (I25.2)	14	3	2 x 10^−36^
Anemia; unspecified (D64.9)	10	3	9 x 10^−12^
Harmful use (F17.1)	10	3	6 x 10^−12^
Urinary tract infection; site not specified (N39.0)	11	4	4 x 10^−10^
Family history of ischemic heart disease and other diseases of the circulatory system (Z82.4)	28	4	2 x 10^−84^
Personal history of long-term (current) use of anticoagulants (Z92.1)	29	4	4 x 10^−92^
Chronic ischemic heart disease; unspecified (I25.9)	19	5	5 x 10^−33^
Procedure not carried out for other reasons (Z53.8)	12	5	1 x 10^−7^
Personal history of diseases of the circulatory system (Z86.7)	19	5	4 x 10^−27^
Angina pectoris; unspecified (I20.9)	20	5	1 x 10^−28^
Atrial fibrillation and flutter (I48)	40	6	1 x 10^−132^
Personal history of diseases of the digestive system (Z87.1)	11	6	1 x 10^−5^
Personal history of long-term (current) use of other medicaments (Z92.2)	21	6	9 x 10^−28^
Asthma; unspecified (J45.9)	14	6	4 x 10^−8^
Chest pain; unspecified (R07.4)	28	6	4 x 10^−53^
Non-insulin-dependent diabetes mellitus w/o complications (E11.9)	13	7	4 x 10^−5^
Atherosclerotic heart disease (I25.1)	23	7	1 x 10^−28^
Diaphragmatic hernia without obstruction or gangrene (K44.9)	15	8	2 x 10^−6^
Diverticular disease of large intestine without perforation or abscess (K57.3)	14	9	2 x 10^−4^
Personal history of psychoactive substance abuse (Z86.4)	28	10	4 x 10^−30^
Essential (primary) hypertension (I10)	61	27	2 x 10^−43^

Diagnoses associated with HCM in the UK Biobank with ICD-10 codes. *P*-values are from chi-square test. (-) *p*-value not calculable due to low total cases and controls.

**TABLE 2:** Relevant biometrics and biomarkers in cases and controls.

Biometric/Biomarker	Cases (*n* = 363)	Controls (*n* = 7260)	P-value
Height (in)	67.3	67.3	NS
Hip (in)	41.4	40.8	2.4 x 10^−3^
Obesity			
BMI (kg/m^2^)	28.7	27.8	1.48 x 10^−4^
Waist (in)	38.3	36.8	2.0 x 10^−7^
Weight (lbs)	185	179	1.6 x 10^−3^
Hemodynamics			
Systolic Blood Pressure (mmHg)	142	143	NS
Diastolic Blood Pressure (mmHg)	81.1	83	2.4 x 10^−3^
Mean Arterial Pressure (mmHg)	101	102	4.0 x 10^−2^
Pulse Pressure (mmHg)	58.4	57.5	NS
Pulse Rate (beats/min)	67.6	69.2	3.1 x 10^−2^
Lipids			
Apolipoprotein A (mg/dl)	142	150	4.9 x 10^−8^
Apolipoprotein B (mg/dl)	98.6	103	3.4 x 10^−4^
Total Cholesterol (mg/dl)	202	217	3.0 x 10^−9^
HDL (mg/dl)	49.9	53.4	8.9 x 10^−7^
LDL (mg/dl)	127	137	1.1 x 10^−6^
Lipoprotein A (mg/dl)	21.2	21.7	NS
Triglycerides (mg/dl)	167	163	NS
Hematologic			
WBC (cells/mm^3^)	7240	6850	4.1 x 10^−4^
Platelet Count (cells/uL)	231000	244000	6.0 x 10^−5^
Mean Platelet Volume (fL)	9.71	9.34	6.9 x 10^−9^
Hemoglobin (g/dl)	14.5	14.5	NS
Hematocrit (percent)	42.2	42.0	NS
Mean Corpuscular Volume (fL)	90.9	91.0	NS
Endocrine			
Glucose (mg/dl)	93.6	93.2	NS
Hemoglobin A1c (%)	5.5	5.5	NS
Renal			
Albumin (g/dl)	4.49	4.53	7.6 x 10^−3^
Blood Urea Nitrogen (mg/dl)	35.8	33.5	2.9 x 10^−3^
Creatinine (mg/dl)	0.825	0.764	6.8 x 10^−4^
Cystatin C (mg/dl)	10.1	9.32	8.1 x 10^−9^
Protein — Total (g/dl)	7.21	7.26	NS
Uric Acid (mg/dl)	6.00	5.54	2.2 x 10^−8^
Microalbumin—Urine (mg/L)	112	36.5	NS

Biometrics and biomarkers associated with HCM in the UK Biobank. *P*-values are from chi-square test. (NS) not significant.

### Identification of Hypertrophic Cardiomyopathy Genetic Variants

Variants of interest met thresholds of a minor allele frequency (MAF) of 0.5% or greater and a *p*-value < 5 x 10^−8^. Identified variants were also assessed for phenotypic associations *via* pheweb.org, a GWAS dataset for electronic health record-derived disease associations from the white British participants in the UK Biobank. PheWeb utilizes a generalized mixed model association test that uses the saddle point approximation to account for case-control imbalance and imputed using the Haplotype Reference Consortium Panel (Michigan Genomics Initiative, 2017).

## Results

A total of 363 individuals (119 females and 244 males) were identified as HCM cases based on ICD-10 diagnostic codes for “obstructive hypertrophic cardiomyopathy” (I42.1) or “other hypertrophic cardiomyopathy” (I42.2) as a primary or secondary diagnosis at the time of this analysis (as of July 2020). This represents a prevalence rate of 1 in 1,342, suggesting disease assignment based on the two ICD-10 codes, while specific, underestimates the HCM prevalence. The majority of cases were self-described as having British ancestry (80.7%). Additionally, 3.3% reported Irish ancestry, 3% reported African ancestry, and 1.9% reported Caribbean ancestry. Baseline characteristics are displayed in Table 3.

**TABLE 3:** Baseline characteristics of HCM cases in UKB.

Baseline characteristics	
Cases (#)	363
Males (%)	67.2
BMI >25 kg/m^2^ (%)	76.6
British ancestry (%)	80.7

Population characteristics of HCM cases identified in the UK biobank.

Common co-diagnosis of HCM cases and controls are shown in Table 1. The 363 cases who were diagnosed with HCM were more likely to be also diagnosed with ventricular tachycardia (ICD-10 Code I47.2) compared to age, sex, ancestry-matched controls (11.6% versus 0.25%, *p*-value < 0.0001 by two-tailed Fisher’s exact test). Cases were also more likely to be diagnosed with syncope and collapse (R55; 16% vs. 3%, *p* < 0.0001), dyspnea (R06.0; 13% vs. 2%, *p* < 0.0001), chest pain (R07.4; 28% vs. 6%, *p* < 0.0001), and atrial fibrillation and flutter (I48; 40% vs. 6%; *p* < 0.0001), consistent with the notion that HCM is associated with increased risks of developing these complications. Not surprising for an inherited condition, 28% reported family history of heart disease (Z82.4) compared to 4% for controls (*p* < 0.0001).

Interestingly, the HCM cases were also more likely to be also diagnosed with chronic ischemic heart disease (I125.9; 19% vs. 5%, *p* < 0.0001), asthma (J45.9; 14% vs. 6%, *p* < 0.0001), non-insulin-dependent diabetes mellitus (E11.9; 13% vs. 7%, *p* = 0.0005), atherosclerotic heart disease (I25.1; 23% vs. 7%, *p* < 0.0001), pure hypercholesterolemia (E78.0; 34% vs. 12%, *p* < 0.0001), and essential hypertension (I10; 61% vs. 27%, *p* < 0.0001).

Overall, individuals with HCM were more likely to be also diagnosed with acute renal failure compared to controls (11% vs. 2%, *p*-value < 0.0001) (Table 1). Consistent with this, mean values for blood urea (35.8 vs. 33.5 mg/dl, *p*-value 1.4 x 10^−4^), creatinine (0.825 vs. 0.764 mg/dl, *p*-value 6.8 x 10^−4^), uric acid (6.0 versus 5.54, *p*-value 2.2 x 10^−8^), and cystatin C (1.01 vs. 0.932 mg/L, *p*-value 8.1 x 10^−9^) were higher in HCM cases than controls, suggesting subtle but consistent diminished renal function in HCM cases (Table 2). There was also a trend toward higher levels of urine microalbumin (112 vs. 36.5 mg/L). Although HCM cases were more likely to be diagnosed with type 2 diabetes mellitus (Table 1), there was no difference in mean serum glucose or hemoglobin A1c levels (Table 2). Lastly, while HCM cases were more likely to be diagnosed with hypercholesterolemia (Table 1), mean total cholesterol and low-density lipoprotein (LDL) levels were statistically significantly lower in HCM cases compared to controls (Table 2).

**FIGURE 1:**
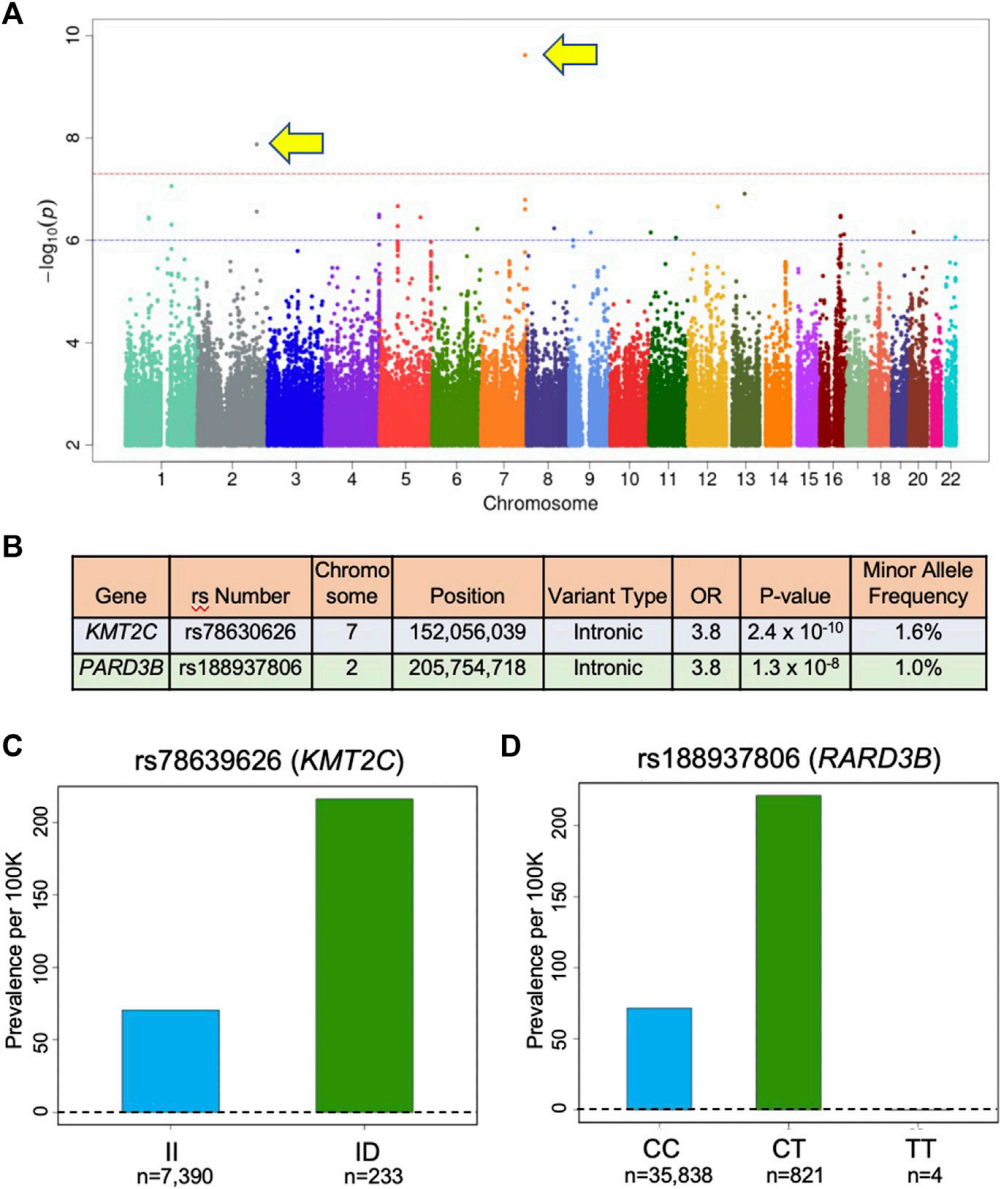
Variants associated with HCM in UK Biobank. **(A)** Manhattan plot of GWAS results (MAF > 0.5) for HCM identifies 2 SNPs associated with HCM diadnosis that are above the red line (p = 5 x 10^−8^) indicating genome-wide significance. In this plot, significance is displayed on the y-axis as −log_10_ of the p-value, with results ordered along the x-axis by chromosomes (each colored bar represents a different chromosome). **(B)** Association results for the SNPs identified in **(A)**. OR is odds ratio and indicates that subjects in the UK Biobank carrying one copy of the minor allele are 3.8 times more likely to have the HCM diagnosis compared to non-carriers. **(C)** Prevalence of HCM in the UK Biobank cohort increases with *KMT2C* variant rs78630626 status (70 per 100,000 for wild-type; 216 per 100,000 for heterozygotes; p-value 1.4 x 10^−10^; OR per D allele = 3.82). **(D)** Prevalence of HCM in the UK Biobank cohort increases with *PARD3B* variant rs188937806 (71 per 100,00 for wild-type; 242 per 100,000 for heterozygotes; 0 per 100,000 for homozygotes; p-value 7.4 x 10^−9^; OR per T allele = 3.85). *KMT2X*: ltsine methyltransferase 2C; *PARD3B*: par-3 family cell polarity regulator beta.

GWAS of variants with MAF of ≥0.5% revealed two variants that were associated with the HCM diagnosis with a *p*-value < 5 x 10^−8^ (Figures 1A,B). First, rs78630626 (MAF 1.08%), an intron in the *KMT2C* gene, encoding lysine methyltransferase 2C, was associated with HCM with the odds ratios of 3.79 and a *p*-value of 2.41 x 10^−10^ (Figures 1A,C). Second, rs188937806 (MAF 1.63%), in an intron in the *PARD3B* gene, encoding Par-3 family cell polarity regulator beta, was associated with HCM with the odds ratio of 3.79 and a *p*-value of 1.33 x 10^−8^ (Figures 1A,D). Of note, no common variants in known sarcomere genes were found to be associated with HCM in this population. Quantile-quantile plots (QQ Plots) are provided in Supplementary Figure S2 to illustrate that the GWAS quality was well controlled.

## Discussion

The approach used in this paper has several limitations. As with any GWAS study, the discovery of novel loci associated with hypertrophic cardiomyopathy does not prove functional causality, and the findings described herein need to be validated by analysis of other databases. Moreover, there are certain limitations inherent to a population study based on ICD-10 codes in comparison to a study dedicated specifically to hypertrophic cardiomyopathy. For example, ICD-10-based studies are limited by the fact that, as in many real-world situations, many diseases and medical conditions are underdiagnosed. Indeed, a total of 363 individuals were identified as HCM cases based on ICD-10 diagnostic codes for “obstructive hypertrophic cardiomyopathy” (I42.1) or “other hypertrophic cardiomyopathy” (I42.2) as a primary or secondary diagnosis at the time of this analysis, represents prevalence rate of 1 in 1,342, indicating disease assignment based on the two ICD-10 codes underestimates the HCM prevalence.

Sarcomeric HCM is an archetypal single gene disorder with autosomal dominant inheritance. However, recent findings indicate that the majority of those diagnosed with HCM do not carry a mutation in sarcomeric genes (sarcomere-negative HCM) (Neubauer et al., 2019). Our GWAS of the UK Biobank cohort identified two variants, one in *KMT2C* and another *PARD3B* that are associated with HCM. Our findings for *PARD3B* are consistent with data published in the PheWeb database (Michigan Genomics Initiative, 2017). The variants we report are common (MAF > 1%), suggesting that they may be genetic contributors to the HCM phenotype, which is increasingly recognized as polygenic and multifactorial (Watkins, 2021). Of note, our GWAS did not reveal association with common variants in any of the sarcomere genes. Presently, we don’t know whether any of the carriers of *KMT2C* and *PARD3B* also harbor rare sarcomere mutations. We attempted to examine the rare sarcomere HCM mutations in the UK Biobank, but at the time of this study, only 40% of the subjects were sequenced. Consequently, there is not enough overlap between carriers of rare pathogenic variants in the sarcomere genes and the reported SNPs in *KTM2C* and *PARD3B* to make a meaningful conclusion. Alternatively, we acknowledge hat association of *KMT2C* and *PARD3B* variants with the ICD-10 codes for HCM diagnosis may not be due to direct association with HCM per se but rather a phenotype resembling HCM (Marian and Braunwald, 2017).

UK Biobank contains a wealth of baseline clinical information of participants, including comorbidities, height, weight, body mass index, and basic laboratory values (Supplementary Table S1) (Bycroft et al., 2018). Several inferences can be made from this information. For example, HCM diagnosis in this cohort is associated with the traditional cardiac risk factors: atherosclerotic heart disease, hypertension, noninsulin-dependent diabetes mellitus, and hypercholesterolemia as well as a host of novel associations. While additional studies are needed to confirm these associations, our findings suggest a possible role of these factors in disease expression of HCM in susceptible individuals. Taken together, our study supports the emerging picture that sarcomere-negative HCM is polygenic and multifactorial (Watkins, 2021). Additionally, we note that despite higher rates of diagnoses of hypertension, noninsulin-dependent diabetes mellitus, and hypercholesterolemia in HCM cases, mean blood pressure, mean hemoglobin A1C and lipid levels are not higher in HCM cases in comparison to age, sex-matched controls (Table 2). This suggests there may be explanations other than biological, such as ascertainment bias in those already diagnosed with HCM. Finally, we found additional biometrics and biomarkers associated with HCM in the UK Biobank (Supplementary Table S1). While we do not yet have a full understanding of these findings, we have included them for future analysis.

Another interesting finding is that in our cohort, HCM cases are associated with a surprisingly high prevalence of atrial fibrillation and flutter (40%), compared to 20% in a case series of 1,558 HCM patients over a 10-year period (Rowin et al., 2017). The reason for this discrepancy is unclear, but we hypothesize that the HCM cases in a cross-sectional population-based cohort like UK Biobank may be different from HCM cases in disease-centric registries.

As with any GWAS study, the discovery of novel loci associated with HCM does not prove a functional association. Nonetheless, our GWAS findings have biological plausibility. For example*, KMT2C* encodes a lysine N-methyltransferase 2C, which is critical for histone H3 lysine 4 (H3K4) methylation necessary for stabilization of gene expression patterns in adult cardiomyocytes and is markedly elevated in hypertrophied cardiomyocytes in transverse aortic constriction (TAC) model and diabetic mice (Stein et al., 2011; Jiang et al., 2017). *PARD3B* encodes Partitioning defective 3 homolog B, also known as PAR3 family cell polarity regulator beta, which is an adapter protein that may play a role in the formation or maintenance of epithelial tight junctions in kidney glomerulus (Koehler et al., 2016) and is found in the urine of preeclampsia patients (Zhao et al., 2011). Interestingly, mean measures of renal dysfunction (serum urea, creatinine, cystatin C and uric acid; and microalbumin) were all elevated in HCM cases, although high urine microalbumin in HCM cases did not reach statistical significance (*p*-value = 0.065) due to wide range of values and relatively few patients who had this measured. Nonetheless, since albuminuria associated is well-known to be associated with left ventricular hypertrophy due to long-standing hypertension (Pedrinelli et al., 1993; Tsioufis et al., 2002; Nabbaale et al., 2015), it is tempting to speculate that subclinical albuminuria, due to intrinsic renal dysfunction, hypertension, or diabetes, contributes to HCM disease manifestation in susceptible individuals.

## Conclusion

Here, using ICD-10 diagnostic codes as a “real world” surrogate for the definitive diagnosis for HCM, we carried out a GWAS using the UK Biobank database and identified two relatively common genetic variants that are associated with the diagnosis of HCM. Both have biological plausibility as modifiers of HCM disease with *KMT2C* previously being associated with hypertrophied cardiomyocytes in a diabetic mouse model and *PARD3B* potentially playing a role in epithelial tight junctions. Compared to the controls, HCM cases were also more frequently diagnosed with several comorbid conditions, such as hypertension, atherosclerotic heart disease, diabetes, and kidney failure, suggesting they may contribute to hypertrophic disease manifestation in genetically susceptible individuals. While the findings must be validated in future studies, our approach supports the feasibility of using real-world data, such as those contained in electronic health records, to gain new insights into the pathophysiology of relatively rare conditions (Hemingway et al., 2018).

## Data Availability

The datasets presented in this study can be found in online repositories. The names of the repository/repositories and accession number(s) can be found in the article/Supplementary Material.
