# Supplementary material for: Identification of Novel Genetic Variants and Comorbidities Associated With ICD-10-Based Diagnosis of Hypertrophic Cardiomyopathy Using the UK Biobank Cohort
Source: Front Genet. 2022 May 24;13:866042. doi: 10.3389/fgene.2022.866042 (PMC9171016; doi:10.3389/fgene.2022.866042)
Supplement: Supplementary file 1 [file Table1.pdf]

Supplementary Table 1. Full Biometrics and Biomarkers in Cases and Controls

Biometric/Biomarker	Cases (n=363)	Controls (n=7260)	*P*-value
Blood Type O	44%	44%	NS
Blood Type A	44%	42%	NS
Blood Type B	9%	10%	NS
Blood Type AB	4%	4%	NS
BMI (kg/m^2)	28.7	27.8	1.48 x10^{-4}
Height (in)	67.3	67.3	NS
Hip (in)	41.4	40.8	2.4 x10^{-3}
Waist (in)	38.3	36.8	2.0 x10^{-7}
Weight (lbs)	185	179	1.6 x10^{-3}
Systolic Blood Pressure (mmHg)	142	143	NS
Diastolic Blood Pressure (mmHg)	81.1	83	2.4 x10^{-3}
Mean Arterial Pressure (mmHg)	101	102	4.0 x10^{-2}
Pulse Pressure (mmHg)	58.4	57.5	NS
Pulse Rate (beats/min)	67.6	69.2	3.1 x10^{-2}
Alkaline Phosphatase (IU/L)	87.5	83.6	1.9E-02
Calcium (mg/dL)	9.54	9.54	NS
Vitamin D (ng/mL)	18.6	19.3	NS
Insulin-like growth factor 1 (nmol/L)	160	162	NS
Sex Hormone Binding Globulin (nmol/L)	46.6	47.1	NS
Testosterone (ng/dL)	257	252	NS
Apolipoprotein A (mg/dL)	142	150	4.9 x10^{-8}
Apolipoprotein B (mg/dL)	98.6	103	3.4 x10^{-4}
Total Cholesterol (mg/dL)	202	217	3.0 x10^{-9}
C Reactive Protein (mg/dL)	85.1	73.8	NS
HDL (mg/dL)	49.9	53.4	8.9 x10^{-7}
LDL (mg/dL)	127	137	1.1 x10^{-6}

Lipoprotein A (mg/dL)	21.2	21.7	NS
Triglycerides (mg/dL)	167	163	NS
WBC (cells/mm^3)	7240	6850	4.1×10^{-4}
RBC (10^6/uL)	4.66	4.62	NS
Platelet Count (cells/uL)	231,000	244,000	6.0×10^{-5}
Platelet Crit (percent)	0.221	0.224	NS
Mean Platelet Volume (fL)	9.71	9.34	6.9×10^{-9}
Lymphocyte Count (10^9 cells/L)	1.96	1.96	NS
Monocyte Count (10^9 cells/L)	540	488	1.6×10^{-6}
Neutrophil Count (10^9 cells/L)	4.51	4.18	3.4×10^{-5}
Eosinophil Count (10^9 cells/L)	0.185	0.180	NS
Basophil Count (10^9 cells/L)	0.0330	0.0336	NS
Nucleated RBC Count (10^9 cells/L)	0.0019	0.0033	NS
Lymphocyte Percent	27.3	29.0	3.2×10^{-5}
Monocyte Percent	7.66	7.28	5.2×10^{-3}
Neutrophil Percent	62.0	60.5	1.4×10^{-3}
Eosinophil Percent	2.57	2.65	NS
Basophil Percent	0.521	0.66	2.4×10^{-2}
Nucleated RBC Percent	0.0307	0.0554	NS
Reticulocyte Percent	1.53	1.35	1.6×10^{-6}
Reticulocyte Count (cells/uL)	71,200	62,400	1.2×10^{-6}
Reticulocyte Mean Volume (fL)	106	106	NS
Sphered Cell Mean Volume (fL)	82.7	82.8	NS
Immature Reticulocyte Fraction	0305	0.292	2.5×10^{-4}
Reticulocyte High Light Scatter Percent	0.476	0.402	3.7×10^{-8}
Reticulocyte High Light Scatter Count (10^{12} cells/L)	0.0221	0.0186	1.7×10^{-8}
Hemoglobin (g/dL)	14.5	14.5	NS
Hematocrit (percent)	42.2	42.0	NS
Mean Corpuscular Volume (fL)	90.9	91.0	NS

Mean Corpuscular Hemoglobin (pg)	31.3	31.4	NS
Mean Corpuscular Hemoglobin Conc. (g/dL)	34.5	34.5	NS
Glucose (mg/dL)	93.6	93.2	NS
Hemoglobin A1c (%)	5.5	5.5	NS
Albumin (g/dL)	4.49	4.53	7.6×10^{-3}
Alanine Transaminase (U/L)	27.2	25.0	NS
Aspartate Transaminase (U/L)	31.0	27.2	2.6×10^{-3}
Bilirubin — Direct (mg/dL)	0.128	0.114	1.8×10^{-4}
Gamma-glutamyl Transferase (U/L)	50.9	40.3	1.5×10^{-2}
Bilirubin — Total (mg/dL)	0.624	0.573	2.9×10^{-3}
Blood Urea Nitrogen (mg/dL)	35.8	33.5	1.4×10^{-4}
Creatinine (mg/dL)	0.825	0.764	6.8×10^{-4}
Cystatin C (mg/dL)	10.1	9.32	8.1×10^{-9}
Phosphorus (mg/dl)	3.60	3.56	NS
Protein — Total (g/dL)	7.21	7.26	NS
Uric Acid (mg/dL)	6.00	5.54	2.2×10^{-8}
Microalbumin – Urine (mg/L)	112	36.5	NS
Creatinine – Urine (mmol/L)	11,100	9,620	NS
Potassium — Urine (mEq/L)	63.3	64.9	NS
Sodium — Urine (mEq/L)	77.8	82.1	NS

Biometrics and biomarkers associated with HCM in the UK Biobank. *P*-values are from chi-square test. (NS) Not significant.
