# Supplementary figures and images for: Identification of Novel Genetic Variants and Comorbidities Associated With ICD-10-Based Diagnosis of Hypertrophic Cardiomyopathy Using the UK Biobank Cohort
Source: Front Genet. 2022 May 24;13:866042. doi: 10.3389/fgene.2022.866042 (PMC9171016; doi:10.3389/fgene.2022.866042)

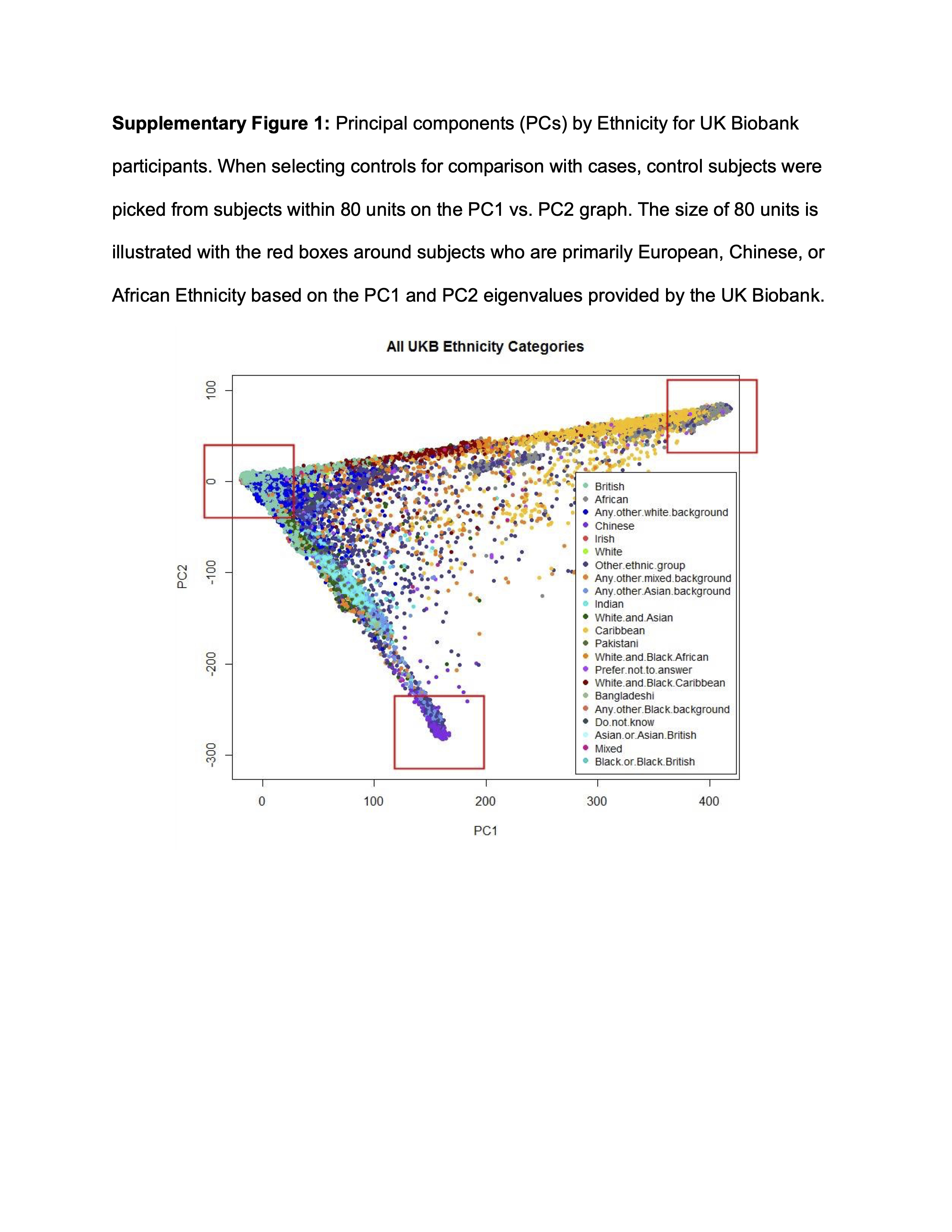

Supplement: Supplementary file 2 [file Image1.JPEG]

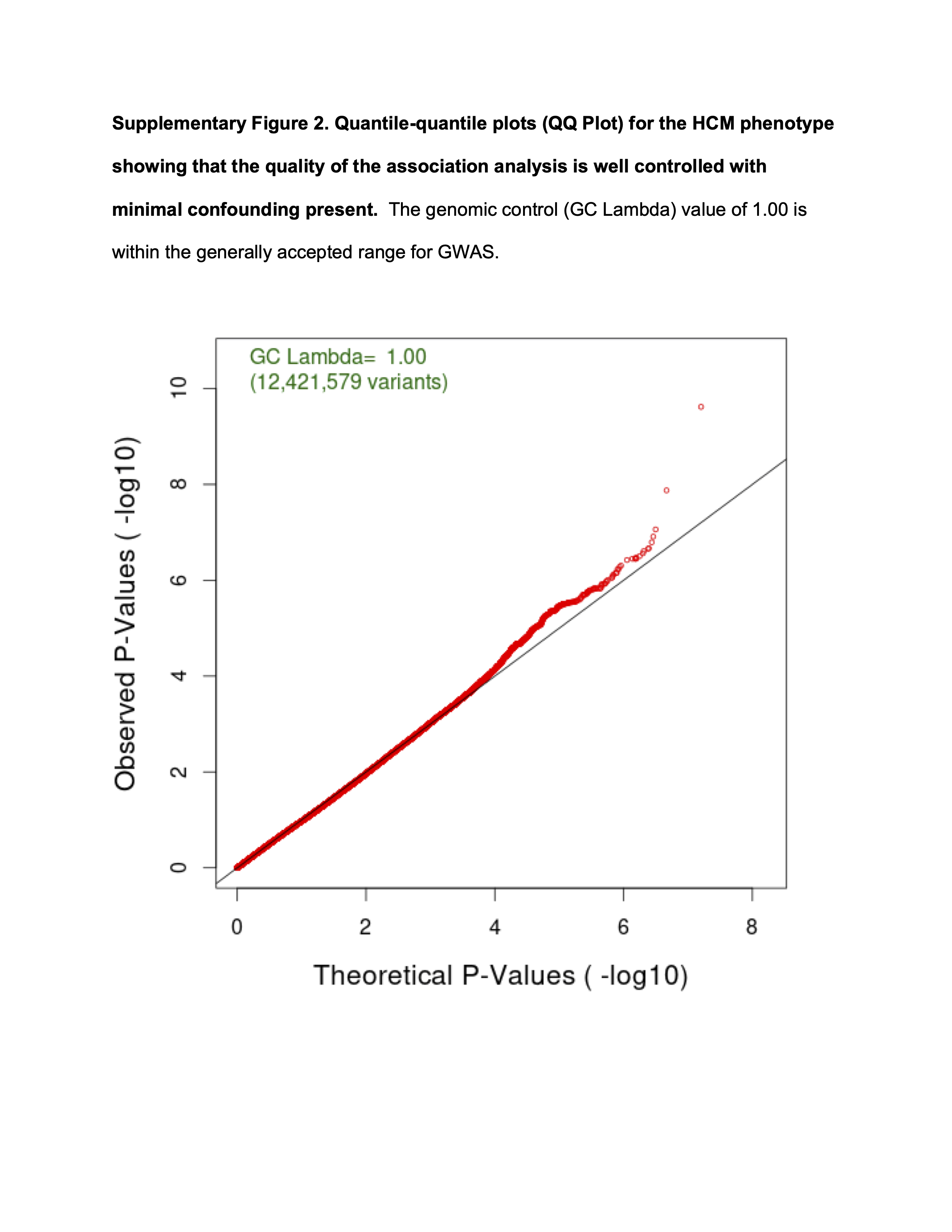

Supplement: Supplementary file 3 [file Image2.JPEG]
